# Green synthesis of metal nanoparticles and study their anti-pathogenic properties against pathogens effect on plants and animals

**DOI:** 10.1038/s41598-024-61920-8

**Published:** 2024-05-18

**Authors:** Osama Usman, Mirza Muhammad Mohsin Baig, Mujtaba Ikram, Tehreem Iqbal, Saharin Islam, Wajid Syed, Mahmood Basil A. Al-Rawi, Misbah Naseem

**Affiliations:** 1 Department of Physics, University of Lahore, Lahore, Pakistan; 2 Department of Physics, University of Barcelona, Barcelona, Spain; 3 Institute of Chemical Engineering and Technology (ICET), University of Punjab, Lahore, Pakistan; 4 Department of Pharmaceutical Sciences, North South University, Dhaka, Bangladesh; 5 Department of Clinical Pharmacy, College of Pharmacy, King Saud University, 11451 Riyadh, Saudi Arabia; 6 Department of Optometry, College of Applied Medical Sciences, King Saud University, Riyadh, Saudi Arabia; 7 Department Chemical Engineering, Beijing Institute of Technology, Beijing, China

**Keywords:** Antipathogens, Bandages, Green synthesis, Nanoparticles, Eco-friendly, Plant pathogens, Biotechnology, Microbiology, Chemistry, Engineering, Materials science, Nanoscience and technology

## Abstract

According to an estimate, 30% to 40%, of global fruit are wasted, leading to post harvest losses and contributing to economic losses ranging from $10 to $100 billion worldwide. Among, all fruits the discarded portion of oranges is around 20%. A novel and value addition approach to utilize the orange peels is in nanoscience. In the present study, a synthesis approach was conducted to prepare the metallic nanoparticles (copper and silver); by utilizing food waste (Citrus plant peels) as bioactive reductants. In addition, the *Citrus sinensis* extracts showed the reducing activity against metallic salts copper chloride and silver nitrate to form Cu-NPs (copper nanoparticles) and Ag-NPs (Silver nanoparticles). The in vitro potential of both types of prepared nanoparticles was examined against plant pathogenic bacteria *Erwinia carotovora* (*Pectobacterium carotovorum*) and pathogens effect on human health *Escherichia coli *(*E. coli*)* and Staphylococcus aureus *(*S. aureus*)*.* Moreover, the in vivo antagonistic potential of both types of prepared nanoparticles was examined by their interaction with against plant (potato slices). Furthermore, additional antipathogenic (antiviral and antifungal) properties were also examined*.* The statistical analysis was done to explain the level of significance and antipathogenic effectiveness among synthesized Ag-NPs and Cu-NPs. The surface morphology, elemental description and size of particles were analyzed by scanning electron microscopy, transmission electron microscopy, energy-dispersive spectroscopy and zeta sizer (in addition polydispersity index and zeta potential). The justification for the preparation of particles was done by UV–Vis Spectroscopy (excitation peaks at 339 nm for copper and 415 nm for silver) and crystalline nature was observed by X-ray diffraction. Hence, the prepared particles are quite effective against soft rot pathogens in plants and can also be used effectively in some other multifunctional applications such as bioactive sport wear, surgical gowns, bioactive bandages and wrist or knee compression bandages, etc.

## Introduction

Fruit and vegetable wastes (FVW) are produced in large quantities in markets and constitute a source of nuisance in municipal landfills because of their high biodegradability^1^. It has been documented that the wastage of fruit contains a major portion of citrus peels, with a world production estimation of 15 × 10^6^ tons/year^2^. The disposal of citrus peels may cause bad environmental impact due to their high chemical oxygen demand (COD) and high biological oxygen demand (BOD). Therefore, the utilization of citrus peels in the area of nanotechnology is expected to minimize problems concerned with their long-term sustainability in the environment and cause pollution^3^. Several studies reported the presence of bioactive compounds, such as phenolic compounds, ascorbic acid and carotenoids in the citrus peel extract. These compounds may act as bioreductants, biodispersents and stabilizing agents during the green synthesis of metal nanoparticles^4^. Among all extracted phytochemicals, the phenolic contents (key compound) play a vital role during the reduction of metallic ions and in converting them into stabilized metallic particles^5^. According to information obtained from recent research, the average total phenolic contents (TPC) were reported between 4.9 and 6.9 mg gallic acid equivalent (GAE)/g fresh weight (FW) citrus peels. Moreover, they also described the yield recovery of phytochemicals as about 73%, obtained in comparison to total solid mass^6^. In fact, the synthesis of metal nanoparticles by synthetic reducing agents has toxic effects not only on human health but also on crop growth and yield (by accumulating in plant tissues, including edible parts). Hence, among all the famous techniques to prepare the metal nanoparticles such as mechanical milling, sputtering, physical method, chemical method and laser ablation etc.^7^. The green synthesis technique is more ecologically satisfactory, nontoxic, perfect, modest, dependable and one stage process. The effects of green synthesized metallic nanoparticles on plant species have been the subject of few studies^8^. A broad exploration has been led to limit the weighty reliance on engineered fungicides for controlling postharvest infections^9^. However, this growing problem necessitates eco-friendly and safe solutions for perishable crops like sweet potato quality^10^. *Pectobacterium carotovorum *(*P. carotovorum*) ubiquitous plant pathogen has been reported frequently with an adverse effect on vegetable host potatoes^11^. A recent study showed the strongest antibacterial effect of copper nanoparticles during the In-vivo and In-vitro analysis of (*P. carotovorum*) effect of copper nanoparticles. On the other hand, the current interest of researchers has also focused on human-infected viruses and bacteria. These viruses and bacteria have a broad effect on hospital-acquired infections. After the outbreak of deadliest pandemic COVID-19, a number of trust worthy sources has declared the use of metallic nanoparticles against SARS-CoV-2^12^. Shahid et al. studied the effect of cuprous oxide nanoparticles coated cotton fabrics against various types of pathogens to deal with hospital-acquired infections. The Cu_2_O coated fabrics showed excellent antibacterial effects against, *E. coli and S. Aureus*. Moreover, various studies demonstrated the In-vivo and In-vitro effect of copper and silver nanoparticles against plant and human pathogenic fungus *Aspergillus niger *(*A. niger*)^13,14^*.* Usha et al. analysed effectiveness of copper oxide nanoparticles coated fabrics against *A. niger* and exhibited about 100% reduction after 48 h of incubation^15^. So, the researcher has been using different types of antimicrobial agents on hospital textiles to reduce the risk factor against infections. However, the use of antimicrobial agents is limited because of their toxicity^16^. So, as an alternative to all aforementioned techniques the use of green synthesis metal nanoparticles is quite well and suitable against several types of pathogens (human and plant infected)^17,18^. Manal et al.^19^ attempted to synthesize Ag-NPs using biological waste material from citrus limon peels. The synthesized Ag-NPs had an average size of 59.74 nm according to DLS measurements and showed strongest antipathogenic effect.

The anti-microbiological activity of copper and silver nanoparticles (prepared from synthetic source) used in plant soft roots has been widely reported. However, the antimicrobial activity of Cu-NPs and Ag-NPs and their role against the pathogens on potato soft roots (from citrus plants waste source has not been reported). Moreover, the same particles were applied on cotton fabric to fabricate the hygienic textiles for human use. The current study was conducted to study the in-vivo and in-vitro potential of green synthesized nanoparticles against bacterial infection in plants and human. To address the aforementioned issues, the waste of citrus fruit *Citrus sinensis* was used as a reducing and stabilizing agent for silver and copper salts. Thus, the coating was done over plant source (potato slices) and cotton bandages. The end applications of the developed textiles are their use as an active agent against soft roots plants and also for the benefit of humans in the fabrication of antimicrobial compression bandages, surgical drapes, panels, covers, shoe mats, scrub suits, table coverings, chair coverings, socks for doctors and patients etc.

## Materials and methods

The peels of fruit (*Citrus sinensis*) were collected from local juice points, a market of Lahore, Pakistan. Copper (II) chloride (CuCl_2_.2H_2_O) and silver nitrate (AgNO_3_) with 99% purity were acquired by Germany’s Riedel–de Haen. While, Merck (Germany) provided 98% pure L-ascorbic acid (used as a capping and reducing agent). None of the compounds were further chemically treated or purified, as they were all of analytical reagent grade. Merck provided LB (Luria–Bertani) agar-based broth and Lennox broth for antibacterial testing. For usage in all of the chemical reactions, every chemical solution was newly synthesized.

### Preparation of plant extract and phytochemical analysis

Experimental research and field studies on plants involving the collection of plant material, were conducted in accordance with relevant institutional, national, and international guidelines and legislation.

**Figure 1:**
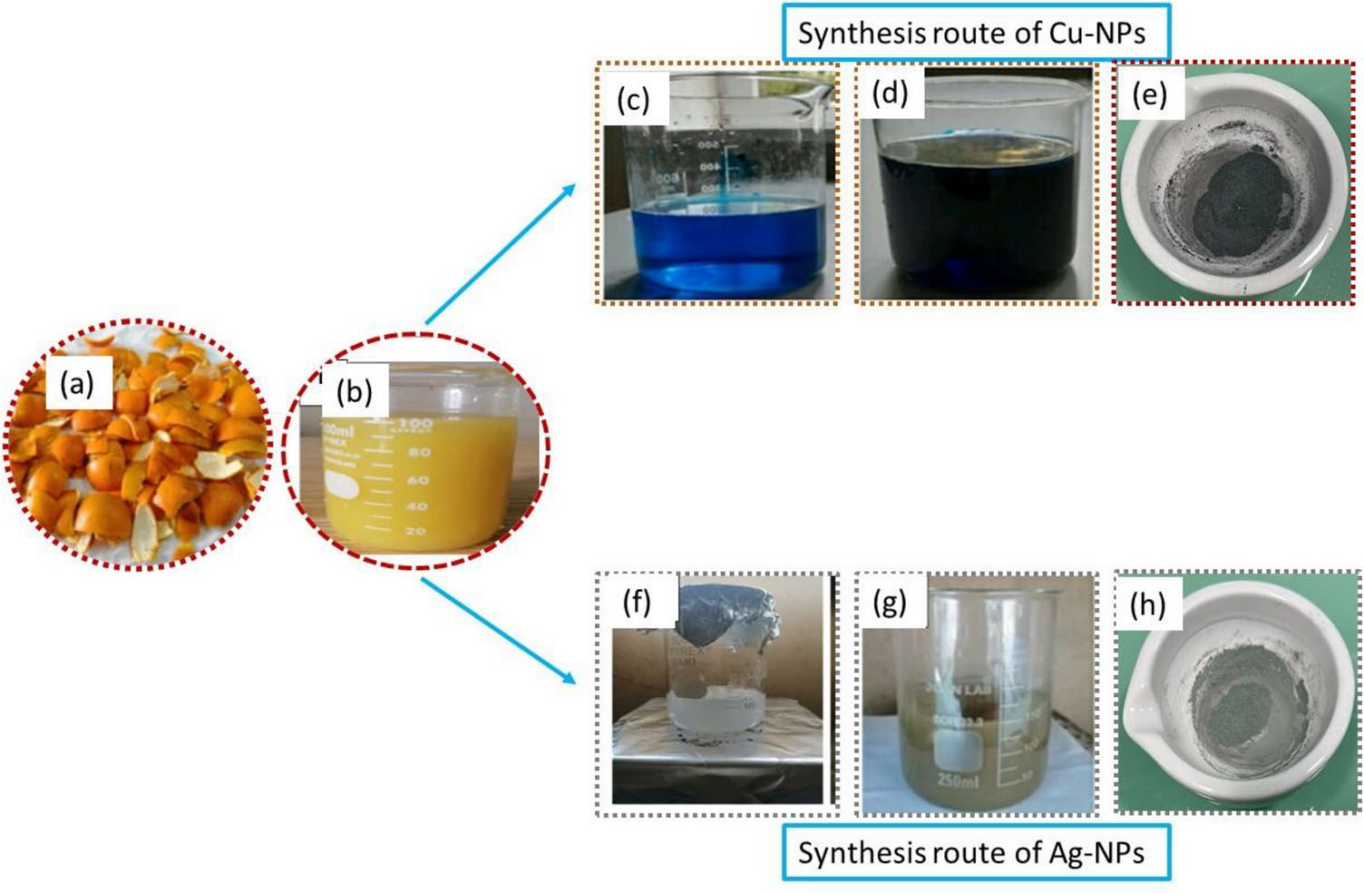
(**a**) *Citrus sinensis* peel flakes, (**b**) extraction through reflux, (**c**) CuCl_2_·2H_2_O solution, (**d**) greenish black solution of Cu-NPs, (**e**) calcinated obtained Cu-NPs, (**f**) AgNO_3_ solution, (**g**) greenish grey solution of silver nanoparticles, (**h**) calcinated obtained Ag-NPs.

The peels of *Citrus sinensis* were properly washed and let to air dry as shown in Fig. 1a. Then, the dry peels were cut into flakes and chopped by using lab scale pestle and mortar. Subsequently, 40 g of chopped peels were added in round bottom flask followed by the addition of 100 mL of distilled water and refluxed at 80 °C for 60 min. Then obtained extraction was heated at 80 °C for 2 h and was left at room temperature to cool down. Afterward, the whole solution was filtered through Whatman filter paper to obtain the fine extract. The obtained extract (Fig. 1b) was then stored in refrigerator at 4 °C for further use. The phytochemical analysis of obtained extract was also analyzed by using standard procedures as described previously^20,21^.

### Green synthesis of Cu-NPs from *Citrus sinensis* fruit peel extract

The hydrated copper chloride (CuCl_2_·2H_2_O) was used as a precursor salt for green synthesis of Cu-NPs. About 30 g/L of CuCl_2_·2H_2_O was mixed by using magnetic stirrer in 100 mL of deionized water (Fig. 1c). The solution was stirred at 60 °C for 15 min followed by the slow addition of prepared extract into the solution. The color of the solution changed from blue to bluish orange after the addition of extract. Then it was stirred further for 30 min. The color of solution turned to greenish black, which justified the synthesis of Cu-NPs (Fig. 1d). Afterward the solution was centrifuged at 8000 rpm for 10 min and placed in furnace for calcination. The blackish green colored copper particles were obtained after the procedure of calcination (Fig. 1e)**.**

### Green synthesis of Ag-NPs from *Citrus sinensis* fruit peel extract

The silver nitrate (AgNO_3_) was used as a precursor salt for green synthesis of Ag-NPs. About 40 g/L of AgNO_3_ was mixed by using magnetic stirrer in 100 mL of deionized water (Fig. 1f). The solution was stirred at 60 °C for 30 min followed by the slow addition of prepared extract into the solution. The color of solution changes to brownish grey, which justified the synthesis of Ag-NPs (Fig. 1g). Then, the solution was centrifuged at 8000 rpm for 10 min. The obtained particles were then placed in furnace for calcination. Greenish grey colored Ag-NPs were obtained after calcination (Fig. 1h).

### Application of prepared particles on substrate (cotton fabric and potato slices)

At first, three different concentrations of each particles were decided (0.25 g, 0.5 g, and 1 g) and dissolved 20 ml of de-ionized water.

**Figure 2:**
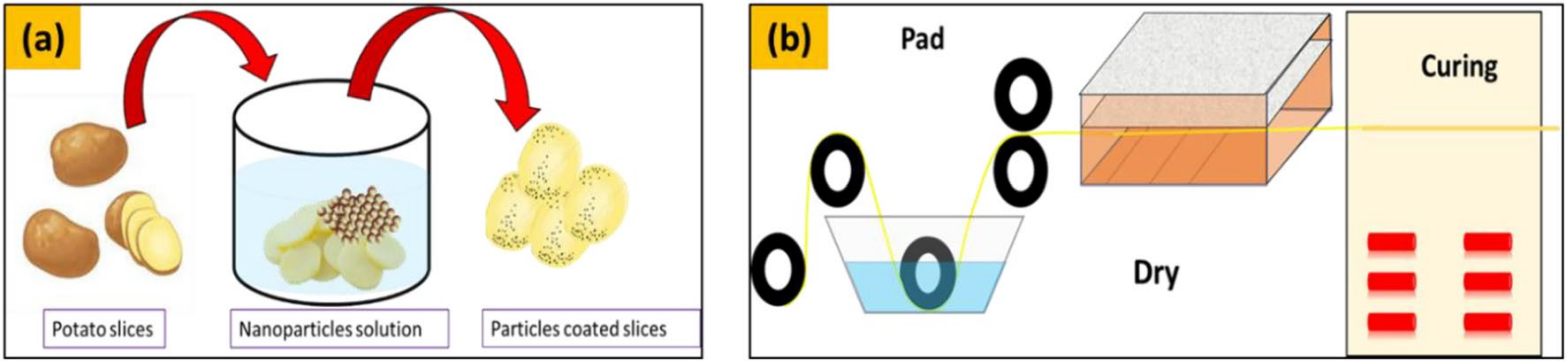
The process of coating the nanoparticles over the (**a**) potato slices, (**b**) fabric structure.

*Application on potato slices* Fresh potatoes having almost same size with (no buds or eyes) were selected to make the slices. All slices were cut in same size and same width (1.5 mm). Then, the slices were transferred into beakers, containing the solutions of different nanoparticles with different concentration (see Table 1). The slices were remained in solutions for whole night to soak the maximum solution contains particles. Then, dried at 50 °C for 20 min in an oven. The schematic for applying the particles over the potato slices is shown in Fig. 2a.

**Table 1:** Design of experiments of developed nanoparticles with different concentrations on different substrates.

No of samples	Particles	Concentration of particles (gram)	Substrate	Code of samples
1	Silver	0.25	Potato soft roots	S1
2	Silver	0.50	Potato soft roots	S2
3	Silver	1.00	Potato soft roots	S3
4	Silver	0.25	Cotton bandage	S4
5	Silver	0.50	Cotton bandage	S5
6	Silver	1.00	Cotton bandage	S6
7	Copper	0.25	Potato soft roots	S7
8	Copper	0.50	Potato soft roots	S8
9	Copper	1.00	Potato soft roots	S9
10	Copper	0.25	Cotton bandage	S10
11	Copper	0.50	Cotton bandage	S11
12	Copper	1.00	Cotton bandage	S12

*Application on cotton bandages* Now, within every solution, 0.5 g of binder was dissolved. Citric acid was used to kept the pH between 5 and 6. After that cotton fabrics with 10 × 10 cm pieces were dipped in the solutions containing different nanoparticles with different concentrations (see Table 1). Subsequently, the cotton cloth was pad in solution and dried for 20 min at 90 °C. The following procedure illustrates the application of particles over the structure of cotton fabric (Fig. 2b). The experimental design for all the developed samples is given in Table 1.

## Testing and characterizations

### Surface characterization of the synthesized nanoparticles

Surface characterizations involved UV–Vis spectroscopy, XRD, and SEM analysis. UV spectroscopy was performed to analyze the absorbance spectra in a wavelength range of 200–1000 nm. The crystalline nature of the nanoparticles was observed by XRD analysis by using Japan Made Model JEOL JDX 3532. Approximately 1 mg of particles powder was used for XRD analysis. The scanning electron microscopy (SEM) was done to observe the surface morphology of the synthesized nanoparticles. The element composition of the biosynthesized nanoparticles was examined by EDX Oxford Company Model INCA 200. Nicolet Nexus 470 spectrometer was used to measure the infrared spectra. The instrument was equipped with an Attenuated Total Reflection (ATR) Pike-Miracle accessory.

### In-vitro and in-vivo testing of synthesized nanoparticles against plant pathogens

The in-vitro antibacterial effectiveness was evaluated by using Disc diffusion method. Plant pathogens bacterial strains (*P. carotovorum*) were used for this purpose. Nutrient agar was used as a culture media. The zone of inhibition against different concentrations of Ag-NPs (0.25, 0.50 and 1.00 g) and different concentrations of Cu-NPs (0.25, 0.50 and 1.00 g) was measured. For in-vivo study, the bacterial strains of (*P. carotovorum*) with constant concentration 50 µl were applied over each sample (copper particle coated potato slice, silver particles coated potato slice and uncoated potato slice). Then, the slices were placed in petri dishes and sealed tightly with parafilm, and incubated for 24–48 h at 35 °C^22^.

### In-vitro testing of synthesized nanoparticles against human pathogens

The in-vitro testing of synthesized nanoparticles was involved antibacterial Qualitative and Quantitative test.

#### Qualitative analysis (zone of inhibition measurement)

##### Bacterial strain preparation

To perform the qualitative analysis, two types of bacterial strains gram positive *S. aureus* (CCM-3953) and gram-negative *E. coli* (CCM-3954) were selected. Each time fresh bacterial suspensions were prepared for cultivating even a single colony in a nutrient bath. The bacterial suspensions were remained in nutrient bath for the duration of the whole night at 37 °C. Prior to start the antibacterial tests the agar plates were prepared carefully by adjusting the sample turbidity at 0.1 with optical density of (OD 600). Subsequently, the Cells were evenly distributed on the agar plates with the help of cotton swab (soaked in the culture media). These plates were used for qualitative analysis of antibacterial testing.

##### Determining zone of inhibition

The qualitative analysis Zone of Inhibition was found against the Ag-NPs and Cu-NPs coated textile samples. The particles coated textile samples, and controlled fabric (not coated with particles) having (6 × 6 mm sq.) dimensions were placed directly on inoculated agar plates. Then whole assembly (samples and inoculated agar plates) were placed at 37 °C for 24 h. Zone of inhibition was analysed around the entire diameter (mm) of the particles coated textile. The calculation was made to measure the area where bacterial growth was inhibited.

#### Quantitative test (reduction factor)

The standard test according to AATCC (100-2004 procedure was used to conduct quantitative measurements). This approach describes the reduction in inoculation bacterial concentration caused by the sample effect by using the reduction factor. The inhabitation in reduction normally calculated by considering the number of surviving bacterial colonies (CFU). Consequently, a comparison of both treated and untreated samples is required (standardized). First, a sample that had been cut into 18 × 18 mm squares was placed in a sterile container for thirty minutes. Next, for each test, a specific bacterial strain (100l) containing 105 CFU/ml was used. After 24 h of incubation at 37 °C in a thermoset, physiological solution was added.

### Antifungal activity

To assess the antifungal properties of the treated samples, the standardized test method AATCC 100-2004 was consistently employed. The specific fungus used in this experiment was *Aspergillus niger*. The antifungal activity was estimated as a percentage change using Eq. (1):

1 $${\text{Percentage reduction}}\;R\left( \% \right) = \frac{{\left( {A - B} \right)}}{A}*100$$

Here, A represents the fungal spore counts for the control fabric, and B represents the spore counts for the treated dyed fabric specimens.

### Antiviral activity

The infectious viral titer was determined using Behrens and Karber method. Briefly, the Vero-E6 cell line cultures were cultivated in Dulbecco’s Modified Eagle Medium (DMEM) supplemented with 9% foetal bovine serum (FBS) and 2% penicillin–streptomycin (PSA). The cultures were infected with the coronavirus and were grown under standard test conditions (24 h at 37 °C in 6% CO_2_) and stored in 96-well plates at a concentration of 2 × 10^5^. To evaluate the viral titer, the supernatant was carefully centrifuged for 30 min at 3700 rpm and temperature of 5–7 °C. The viral titer in the developed cell lines was calculated using the Behrens and Karber’s method. For Next, vials containing the fabric samples were filled with a 20 mm × 20 mm section of fabric. The filter was used to eliminate any extractable viral loads in the channels after passing a 100 µl infection rate through the test sample. The infectious coronavirus was diluted from 101 to 108 repetitions. Vero-E6 cell cultures were grown for three days under optimal conditions at 37 °C and 6% CO_2_ after injecting each serial dilution. The titers of the corona virus in the cultured cell lines were determined the same method.

### Statistical analysis

The extent of relationships between dependent and independent variables was determined using the ANOVA tool by MINTAB software.

## Results and discussion

The nanoparticles synthesized by green method were characterized by various characterization techniques.

### Phytochemicals screening analysis

At first, the screening of extracted phytochemicals was done by using the standard methods obtained from various studies. The screening showed the presence of all active compounds necessary for the reduction, dispersion and stabilization of metal ions. The obtained extract from the citrus leaves were contained the phytochemicals such as flavonoids, phenols, steroids, glycosides and terpenoids. Several studies have already been reported about the presence of alkaloids, saponins, tannins, sterols and flavonoid in citrus extract. Flavonoids are most desiring and key component among all phytochemicals. In fact they played a dual role, as a reducing agent and as an antibacterial agent due to their inherent antipathogenic characteristics. Flavonoids also have antioxidative, cytotoxic, chemopreventive, and antiprnoliferative properties^23^. The list of extracted phytochemicals in accordance with the relevant studies is given in Table 2.

**Table 2:** Phytochemicals present in leaf of *Citrus sinensis.*

	Phytochemicals	Methods	Leaves of *Citrus sinensis*	Refs.
1	Flavonoids	Lead acetate test, alkaline reagent test, ferric chloride test	+	^24^
2	Phenols	Ferric chloride test	+	^21^
3	Steroids	Liebermann-Burchard’s test	+	^21^
4	Terpenoids	Copper acetate test	+	^25^
5	Tannins	Ferric chloride test, gelatin test, lead acetate test, alkaline reagent test	−	^24^
6	Glycosides	Killer-Killiani test	+	^21^
7	Alkaloids	Wagner’s test, mayer’s test, hager’s test, dragenfdroff test	+	^24^
8	Carbohydrates	Molisch’s test, benedict’s test, fehling’s test	+	^25^

Present +, Absence −

### Distribution of particle sizes, polydispersity index and zeta potential

**Figure 3:**
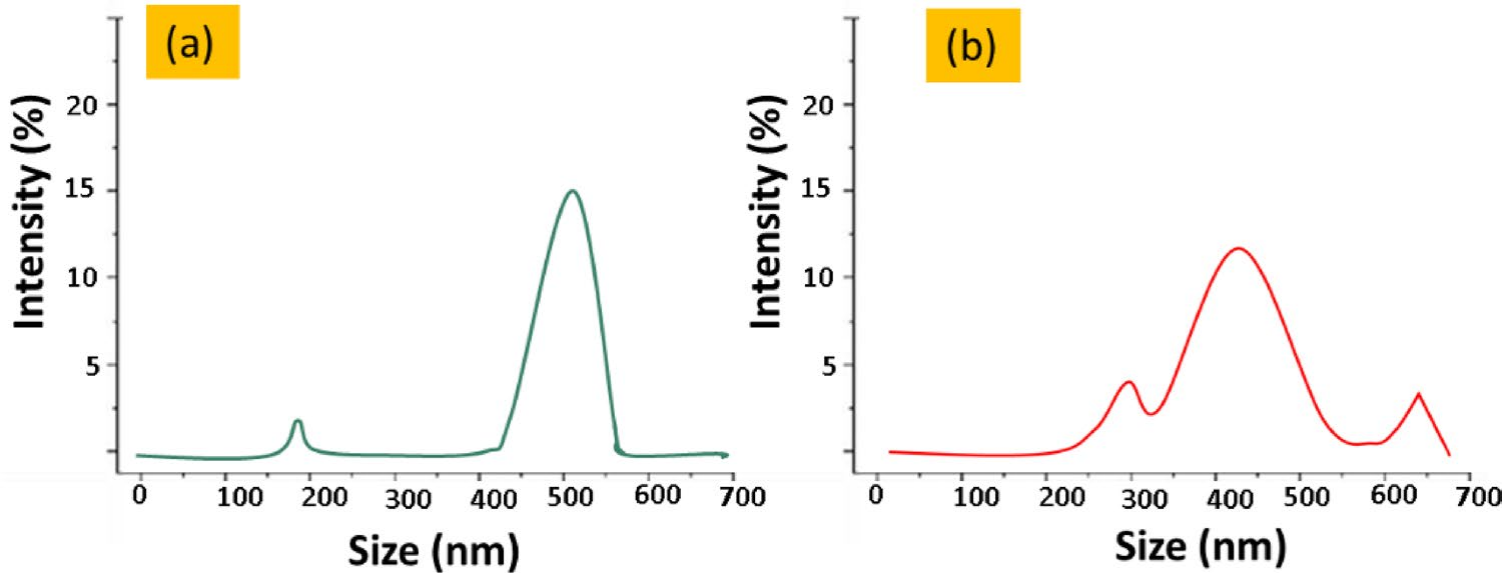
The particle size distribution of (**a**) Cu-NPs and (**b**) Ag-NPs.

The molecule size was calculated by using DLS methods, based on the Brownian motions of the particles. Figure 3a,b respectively demonstrate the average particle size distribution for Cu-NPs and Ag-NPs. The sizes of particles were varying in size from nano meter to milli meter range in a multi-modal manner. The average size of copper and silver particles were notices about 510 nm and 470 nm respectively, at a zeta potential of − 41.6 mV and − 30.6 mV respectively. The Zeta potential values showed the colloid stability of both types of nanoparticles, as these values were > ± 30 mV^22^.This ensured that the particles were evenly distributed throughout the suspension and had a high negative potential from the Nano meter to the micro range. The stability of particles is well analyzed by zeta potential, while the particle size distribution in nano sciences is more articulate with polydispersity index (PDI) values. The polydispersity Index (PDI) of Ag-NPs and Cu-NPs was noted about 0.312 and 0.258 respectively. The values show that the synthesised particles are highly polydisperse^26^.

### Surface characterizations

**Figure 4:**
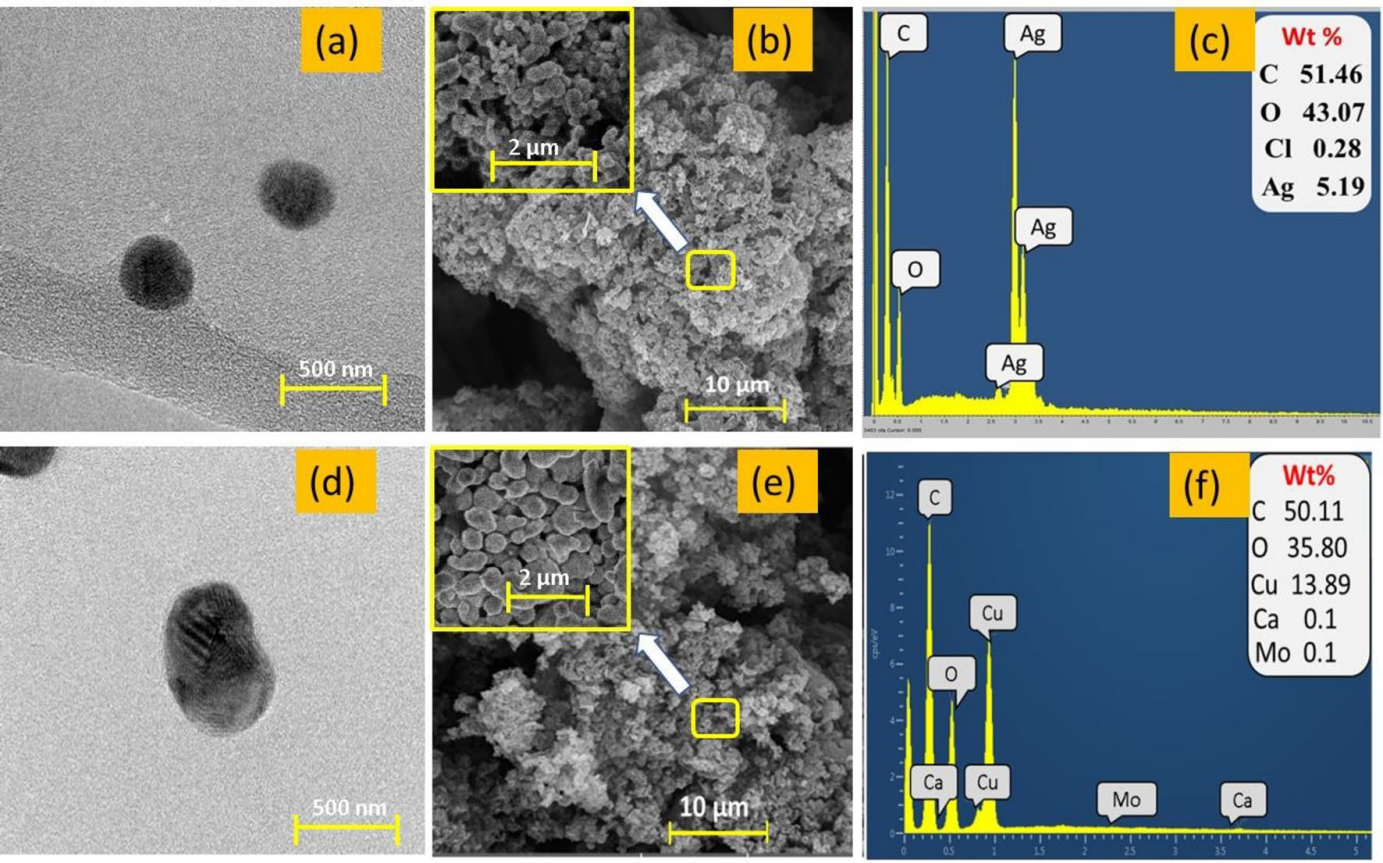
(**a**–**c**) TEM analysis, SEM analysis and EDX spectra of silver particles (**d**–**f**) TEM analysis, SEM analysis and EDX spectra of copper particles.

The surface morphology of synthesized copper and silver nanoparticles was examined using scanning electron microscopy (SEM). The external morphological investigation through SEM revealed the formation of Ag-NPs, and Cu-NPs at the nano to micrometric scale. The rough surface and random clusters with cylindrical form for both types of particles was observed. The tiny agglomerations with constant repetition and even deposition was also noticed. SEM images also revealed the irregular spherical morphological features of the biosynthesized nanoparticles as shown in Fig. 4b,e. However, the sizes of all particles were noticed within the nanometric sale by zeta size analysis as described in the previous Section “Distribution of particle sizes, polydispersity index and zeta potential”. More SEM images at higher magnification in their respective box are added to deeply analyze the connectivity of particles. Which showed the dense coating and homogeneous connectivity between the particles over the fabric structure. However, they were observed within the range of nanometric scale. Moreover, the TEM analysis were also performed to better clarify the sizes and morphologies of nanoparticles as shown in Fig. 4a,d. The TEM analysis estimates the sizes of copper and silver particles between 400 and 500 nm and report the morphologies nearly spherical. As aforementioned, SEM analysis shows the nanoclusters of particles, ranging in 500 nm. While during TEM analysis, it seems that the separate particles of copper and silver are broken parts from their respective clusters^27^.

Additionally, the elemental compositions of Cu-NPs and Ag-NPs were also estimated to found the amount of metal in percentage. The elemental composition of EDX was estimated using spectrum analysis also uncover additional information about the makeup and components of particles as shown in Fig. 4c,f. Except of oxygen and carbon, some other peaks of impurities in least amount were also noticed such as Ca, Mg, and Cl. The existence of trace elements with low quantities is normal behavior during elemental analysis^28^.

### Justification for the formation of copper and silver particles

The UV–Visible spectroscopy was conducted to justify the synthesis of Cu-NPs and Ag-NPs.

**Figure 5:**
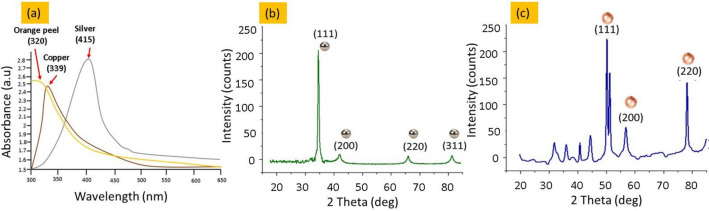
(**a**) UV–Vis Spectrum of the orange peels extract, synthesized copper and silver nanoparticles, (**b**) XRD peaks of silver nanoparticles and (**c**) XRD pattern of Cu-NPs.

The aqueous solution of nanoparticles was mixed with constant ratio (1:2) in distilled water. Subsequently, were mixed well and prepared for UV analysis. The UV spectrum obtained from the synthesized nanoparticles were noticed at 339 nm and 415 nm for copper and silver respectively. While the UV–Vis absorbance spectrum of orange peels extract was notes at λ max was noted 320 nm due to the respective signal of phenolics groups Fig. 5a^29^. In fact, the significant shifts in values and peaks of metal particles as compared to orange extract values is due to the changes in the morphology, size or surface microstructures of silver and copper nanostructures.

Moreover, the XRD analysis was done to justify the formation of crystalline nature of coper and silver particles. The phase purity of manufactured Ag particles was confirmed by exact indexing of all the peak intensity to the silver structure, as shown in Fig. 5b. The four peaks for Ag-NPs appeared at 2 values of 77.5, 64.5, 44.3, and 38.1, respectively. These peaks were attributed to cubic-shaped diffraction planes (3 1 1), (2 2 0), (2 0 0), and (1 1 1), according to data from the International Diffraction Centre (data number JCPDS 04-0783 card)^30^. No significant peaks were observed for other impurities, such as silver oxide. Figure 5c represents the XRD spectrum of Cu-NPs. A precise identification of every diffraction peak to the copper structure reveals the elemental composition of Cu particles. Cu diffraction planes (2 2 0), (2 0 0), and (1 1 1) are characterized by the occurrence of Cu diffraction pattern (2θ) at 74.2, 59.5, and 43.3°. From either the presence of peak position, the copper particles crystalline structure was investigated. Because no distinct impurity peaks were found, other than the development of the Cu_2_O peak (2θ) at 38°, the widening of the peaks instead indicated the synthesis of Cu particles at the nano range, respectively^31^.

**Figure 6:**
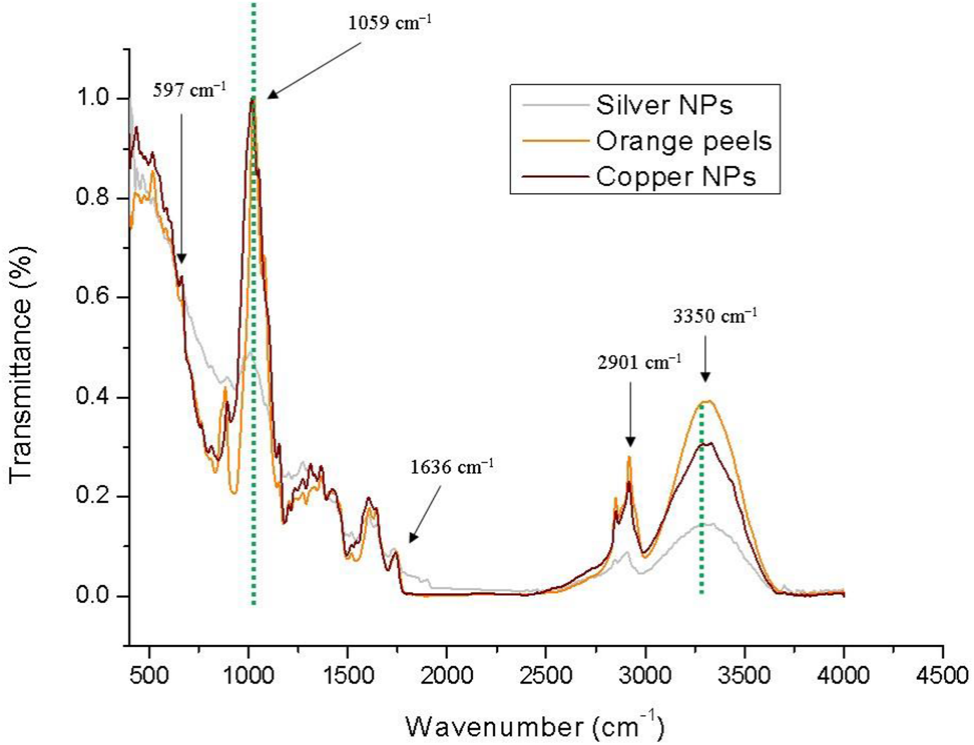
The FT-IR spectra of orange peels extract, copper particles and silver particles coated fabrics.

The extract of orange peels was used as a bio-reductant to synthesized the nanoparticles of copper and silver. Therefore, FT-IR spectroscopy was employed to confirm this reduction process. An analysis of the FT-IR spectroscopy analyzed the presence of functional groups on green synthesized silver and copper particles. The Fig. 6 is illustrating the respective FT-IR spectra of orange peels extract coated cotton fabric, copper particles coated and silver particles coated fabrics. The absorption peaks around 2950, 3331, 2115, 1636, and 597 cm^−1^. On 2950 are C–H stretching vibration absorption peaks in cellulose. While, the broad absorption band on 3670 cm^−1^ corresponds to the O–H stretching frequency, while at 1636 cm^−1^ depicts the C=O stretching of the carbonyl group. The peak 1059 cm^−1^ was noted due to the to the link of alcohols or esters (C–O–H or C–O–R)^32^.

### The in-vitro analysis of synthesized nanoparticles against plants pathogens

**Figure 7:**
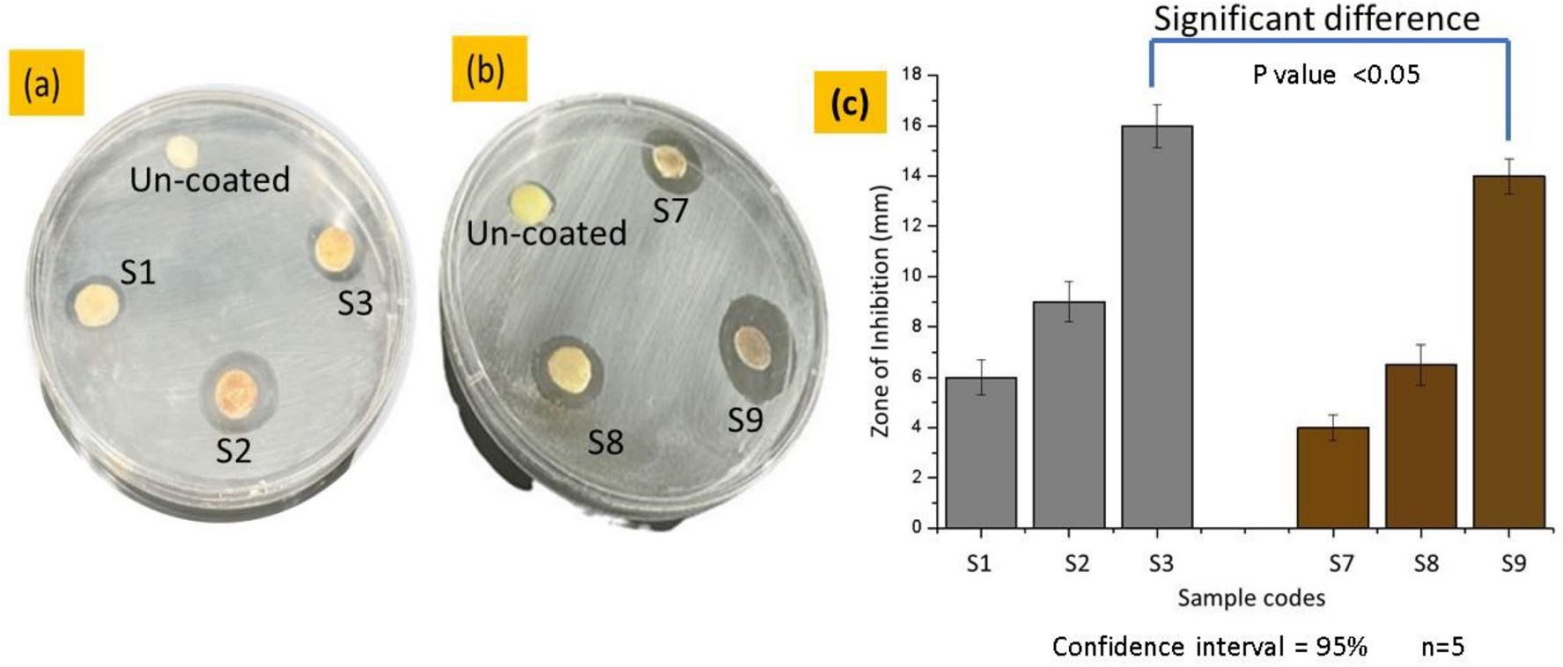
Growth inhibitions in response to (**a**) Cu-NPs and (**b**) Ag-NPs against plant pathogens (*P. carotovorum*) and (**c**) graphical representation of zone of inhibition values for samples coated with silver and copper particles.

The In-vitro analysis of the biosynthesized silver and copper nanoparticles was carried out by using disc diffusion method. The bacterial strains *P. carotovorum,* was used to check the antibacterial potential of biosynthesized Ag-NPs and Cu-NPs. Nutrient agar was used as a culture media. Different dilutions of the synthesized nanoparticles were used to analyze zones of inhibitions against the bacterial strains. The zone of inhibition against different concentrations of silver particles coated potato slices samples S1 (0.25), S2 (0.50) and S3 (1.00 g) and different concentrations of copper particles coated potato samples S7 (0.25), S8 (0.50) and S9 (1.00 g) are shown in Fig. 7a,b.

The silver particles coated potato sample S3 (1.00 g) showed the maximum zone about 16 mm. In a similar study, the antibacterial activity was noted against *P. carotovorum* by silver nanoparticles. Their results revealed that the Ag-NPs showed largest inhibition zone of about with the 14.33 mm^33^. While the copper particles coated sample S9 (1.00 g) showed the zone about 14 mm. It means the silver particles are little bit more effective as compared to copper particles. Azam et al., conducted a comparative analysis of copper and silver particles against different pathogens. Where, silver particles coated substrate showed better performance as compared to copper particles^34^. However, the overall efficiency of both particles is quite effective against bacterial strains *P. carotovorum.* Figure 7c is showing the bar graphs with standard errors against inhibition zone of all silver and copper particle coated potato samples. The group S3 and S9 contains the zone of inhibition values against Ag-NPs and Cu-NPs coated potato samples. During the observation the tail of error bar of silver particles coated sample S3 (1.00 g) group is not coinciding with the head of the error bar of copper coated S9 sample. It means there is a insignificant difference between two groups, which implies that silver and copper particles coated samples have different zone of inhibition range. The ANOVA analysis at 95% confidence interval was applied. The *P* value for group control sample and copper coated sample was calculated as 0.047 which is *P* < 0.05. Hence the *P* value was less than 0.05 which means null hypothesis is insignificant and there is significant difference between the values of sample group S3 and S9. So, there is significant difference between the inhibition Zone against Ag-NPs and Cu-NPs.

### The in-vivo antagonistic potential of nanoparticles against plant pathogens

**Figure 8:**
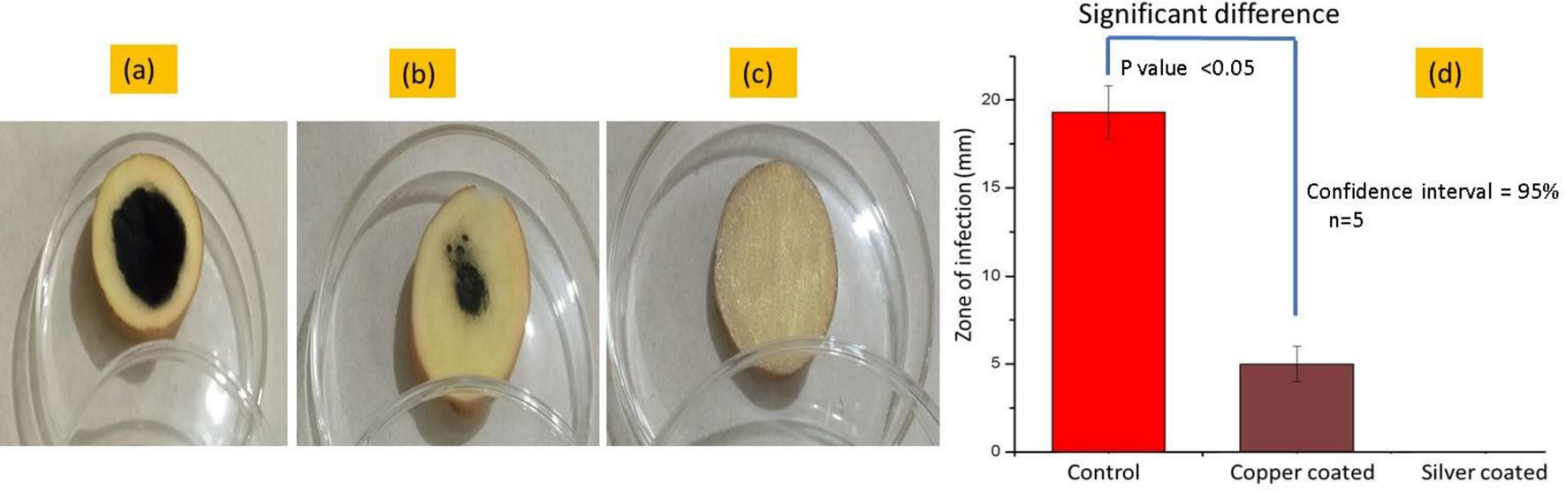
Potato slices with zone of infection caused by *P. carotovorum* (**a**) uncoated sample, (**b**) coated with copper particles and (**c**) coated with silver particles and (**d**) bar graphs showing the values of their respective zone of infections.

The potato slices coated with different concentrations of silver particles coated potato slices samples S1 (0.25), S2 (0.50) and S3 (1.00 g) and different concentrations of copper particles coated potato samples S7 (0.25), S8 (0.50) and S9 (1.00 g) were also subjected to In-vivo Antagonistic potential. The bacterial strains of (*P. carotovorum*) with constant concentration 50 µl were applied over each sample (silver particle coated potato slice S3 (1.00 g), copper particles coated potato slice S9 (1.00 g) and uncoated potato slice). Afterwards, the slices were put in petri plates and covered with para-film and incubated for 24–48 h at 35 °C. The diameter of the infectious zones with measured values are shown in Fig. 8a–c and their values of infection in graphical representation are shown in Fig. 8d respectively. No or almost zero zone of infection was observed on potato slice coated with silver particles S3, while the potato slice coated with copper particles S9 showed slight zone of infection. The reason we have already described in previous section (see section In-Vitro antagonistic), where silver particles proved more effective against pathogens as compared to copper particles. Furthermore, the clear and large zone of infection was seen on uncoated potato slice. It means nanoparticles are quite effective against the bacterial strains of (*P. carotovorum*)*.* The tail of error bar on control sample group is not coinciding with the head of the error bar of copper coated error bar. It means there is significant difference between two groups, which implies that the coating of copper over the potato sample significantly reduce the infection against the bacterial strains of (*P. carotovorum*)*.* Also, there is no group in the place of silver coated sample (as there was no zone of infection). So, A huge difference between each group is present and promotes to the significant difference. The ANOVA analysis at 95% confidence interval was applied. The *P* value for group control sample and copper coated sample was calculated as 0.029 which is *P* < 0.05. Hence the *P* value was less than 0.05 which means null hypothesis is insignificant and there is significant difference between the control and copper coated sample. So, there is significant difference between the infection zones.

Moreover, the area of slice without infection in percentage (area free from bacterial attack) was also calculated by using the following Eq. (2).

2 $${\text{Area}}\;{\text{without}}\;{\text{infection }}\left( {\% {\text{age}}} \right) = \frac{Outer\; Zone - Inner\; Zone}{{Outer \;Zone}}\; \times \;{100}$$

The calculated values of percentage of clear area from bacterial strains are given in Table 3. The silver particles coated samples showed almost 100 percent clear area (i.e. not a single spot or colony of bacterial strain). While there was 87 percent bacterial free area was calculated for potato slice coated with copper particles. In case of potato slice having no coating of particles showed less bacterial free area, which is only 54.5 percent.

**Table 3:** Calculated values of zone of infection, and area without infection in percentage with sample codes.

No of samples	Particles	Concentration of particles (gram)	Code of samples	Potato slice size (mm)	Zone of infections (mm)	Area without infection (%age)
1	Control	0	S0	42.4	19.3	54.5
2	Silver	1.00	S3	44.2	0	100
3	Copper	1.00	S7	38.6	5	87.0

### In-vitro potential of synthesized nanoparticles against human pathogens

To evaluate the effectiveness of the coated textiles for antibacterial properties, both qualitative and quantitative test were conducted.

#### Reduction factor (quantitative test)

**Figure 9:**
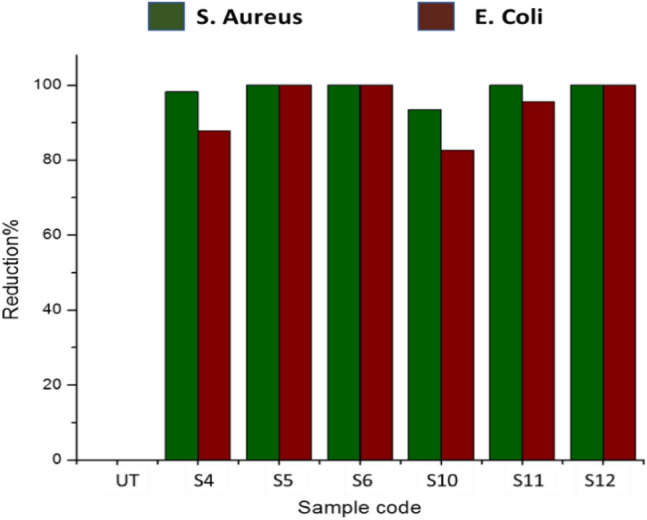
Antibacterial activity in terms of log CFU/ml (left) and percentage reduction (right) of fabrics treated with silver and copper nanoparticles and untreated cotton fabric.

The quantitative technique according to AATCC-100 method was used to measure bacterial resistance against *S. aureus* and *E. coli* strains. Figure 9 presents the reduction percentage of the bacterial cultures on the treated and untreated textile samples. The effectiveness was checked against different concentrations of silver particles coated fabric samples S4 (0.25 g), S5 (0.50 g) and S6 (1.00 g) and different concentrations of copper particles coated fabric samples S10 (0.25 g), S11 (0.50 g) and S12 (1.00 g). The control sample was ineffective against the tested microorganisms. All of the treated samples showed higher reduction in percentage against both type of bacteria, as the amount of Cu-NPs (from 0.25 to 1 g) and Ag-NPs (from 0.25 to 1 g) on the fabric increased. The maximum reduction about 99.99% was found in case of both types *S. aureus* and *E. coli* bacterial colonies. It was noteworthy that all fabric samples coated with silver (S4 to S6) and copper particles (S10 to S12) showed about 99.99% reduction against *E. coli* as the compared to *S. aureus*. It means that *E. coli* is more susceptible to metal particles than to *S. aureus*. The reason is *E. coli* can survive less in open environment (cause less infections), easily vulnerable to antibiotics due to its interactive membrane. While the *S. aureus* can stay longer and resist a range of antibiotics and cause serious infections and leads to different physical rheological responses^35,36^.

**Figure 10:**
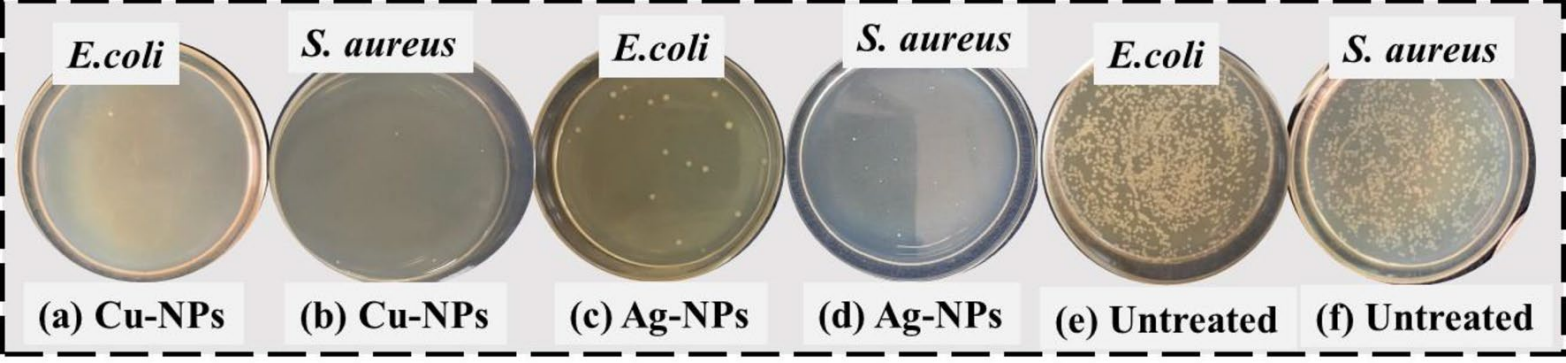
Images of concentration of bacterial growth for the (**a**,**b**) copper particles, (**c**,**d**) for silver particles and (**e**,**f**) for untreated fabrics.

Figure 10 provides additional evidence for the aforementioned trend by displaying the development of bacteria concentrations for silver coated fabric sample S6 (1 g of particles) and copper coated fabric sample S12 (1 g of particles). The untreated cloth was shown to be inefficient against bacterial growth when compared to textiles coated with copper and silver particles. The copper and silver particles coated samples showed maximum reduction in bacterial colonies against both type of pathogens (*E. coli and S. aureus*). At greater concentrations, colony reductions showed a substantial increase, with more than 99 percent efficiency for both species of bacteria^37^.

The silver particles coating on cotton fabric in present study showed better performances compared to previously reported study, where the incorporation of Ag NPs into cotton fabrics using UV photo-reduction was performed^38^. Their results also support the declaration about increase in concentration has direct relation on the reduction of antimicrobial activity of *E. coli*. Several researches have been conducted for the analysis of antimicrobial activities of Ag-NPs coated bandages, and their impact on bacterial strains. The exact mechanism of reduction or inhibition of bacteria growth is still partially understood. In fact, some vibrant concepts involve the release of Ag^+^ and interaction with cell walls. Moreover, these silver ions can also interact with released -SH groups from cellular excretions; and leads to further inactivation of proteins. Hence, the released Ag^+^ ions may again combine another protein when the current protein is decomposed. The silver ions also expediate the production of oxidized radicals; which can penetrate easily into cell wall structure^39^.

#### Zone of inhibition test (qualitative measurements)

**Figure 11:**
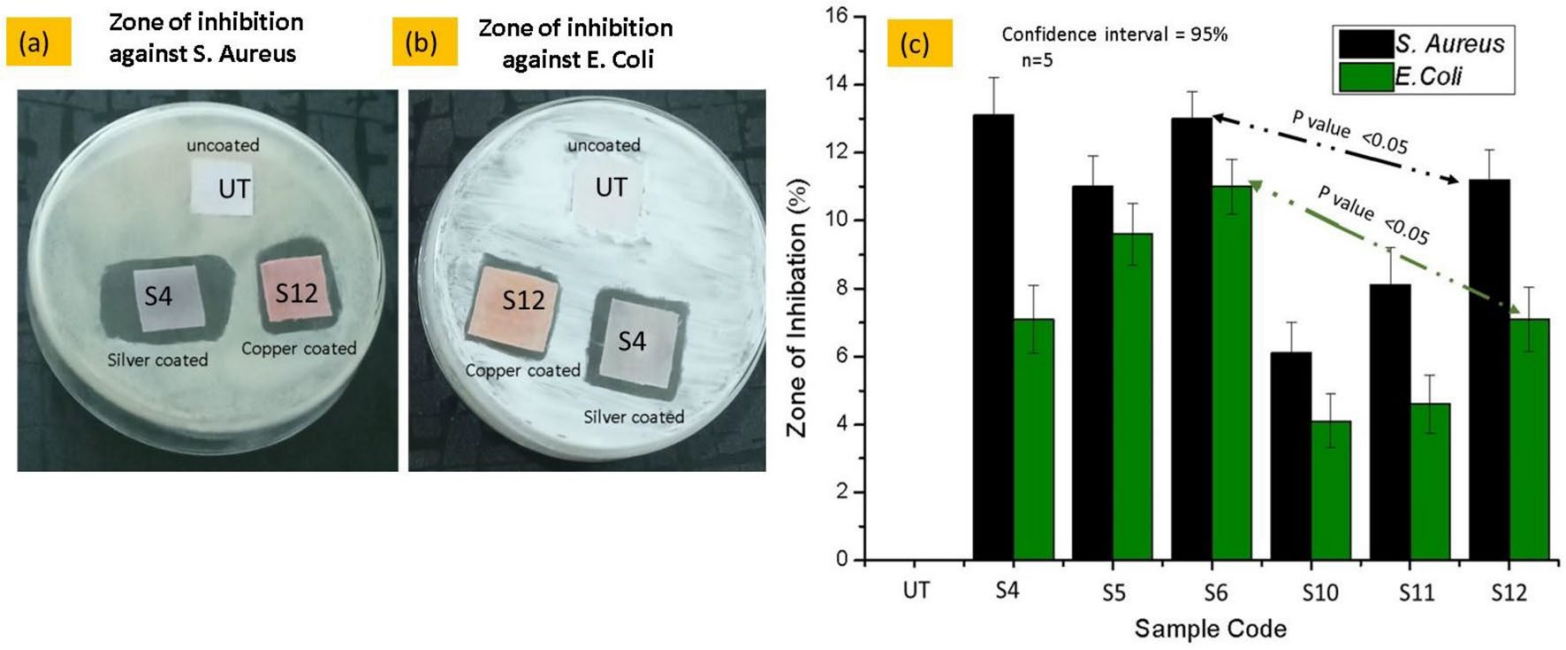
Inhibition zones (**a**) against *S. aureus*, (**b**) *E. coli.*, (**c**) graphical representation of Zone of inhibition around all samples.

Zone of inhibition test was also used to assess the samples antibacterial abilities. Both Gram-positive (*S. aureus*) and Gram-negative (*E. coli*) bacteria were incubated for 24 h at 37 °C in the dark, all fabric samples had distinct inhibitory zones, as seen in Fig. 11a,b. The effectiveness was checked against different concentrations of silver particles coated fabric samples S4 (0.25 g), S5 (0.50 g) and S6 (1.00 g) and different concentrations of copper particles coated fabric samples S10 (0.25 g), S11 (0.50 g) and S12 (1.00 g). The textiles treated with silver nanoparticles (S4 to S6) had the greatest antibacterial zones against the strains of *S. aureus and E. coli*, whereas the fabrics treated with Cu-NPs (S10-S12) exhibited a smaller zone of inhibition. The average values were computed by conducting three readings of each sample. The outcomes showed that the free-standing nature of the copper and silver particles led to considerable disinfection of both bacterial strains, where *S. aureus* demonstrating more sensitivity than *E. coli.* As an illustration, using copper and silver particles raised the area of inhibition for *E. coli* from 4.5 to 10.7 mm, while increasing the area of inhibition for *S. aureus* from 6.5 to 14 mm as shown in Fig. 11c. It should be noted that the increase in inhibition zone with the increase in the concentration of nanoparticles had already been discussed in some previously published research works^30^. The combination of physical and chemical action of bacteria with particles is assumed to be the cause of coated textiles antibacterial properties. Through endocytosis procedures, the nanoparticles are absorbed by the cells. Ionic species are produced inside the cells during the nanoparticles degradation, increasing the cells ability to absorb ions^40^. Silver is showing good antimicrobial ability. In fact, the less antipathogenic effect of copper coating over the substrate as compared to silver was due to the less stability of copper. The similar effect of antimicrobial effectiveness was observed in some relevant studies. Where the in-situ deposition of copper and silver particles was performed to achieve the electrical conductivity and antimicrobial effectiveness. The reason for low electrical performance and bioactive performance was due to the susceptibility of copper particles to oxidation and carbonization^41^.

The group S6 contains the Ag-NPs coated fabric samples showing the zone of inhibition values against *S. aureus, E. coli,* whereas group S12 contains the Cu-NPs coated fabric samples showing the zone of inhibition values against *S. aureus, E. coli.* While comparing the zone of inhibition, values against *S. aureus* were between S6 and S12. The tail of error bar of silver coated S6 sample group (zone of inhibition around *S. aureus* black bar) is not coinciding with the head of the error bar copper coated S9 sample of (zone of inhibition around *S. aureus* black bar). It means there is significant difference between two groups, which implies that silver and copper particles coated samples have different zone of inhibition range. The *P* value between these two groups was observed as 0.037 which is *P* < 0.05. Hence the *P* value was less than 0.05 which means there is significant difference between silver coated S6 sample group (zone of inhibition around *S. aureus* black bar) and copper coated S9 sample of (zone of inhibition around *S. aureus* black bar).

In the same way, while comparing the zone of inhibition values against *E. coli* between S6 and S12. The tail of error bar of silver coated S6 sample group (zone of inhibition around *E. coli* green bar) is not coinciding with the head of the error bar copper coated S9 sample of (zone of inhibition around *E. coli* green bar). It means there is significant difference between two groups, which implies that silver and copper particles coated samples have different zone of inhibition range. The *P* value between these two groups was observed as 0.029 which is *P* < 0.05. Hence the *P* value was less than 0.05 which means there is significant difference between silver coated S6 sample group (zone of inhibition around *E. coli* green bar) and copper coated S9 sample (zone of inhibition around *E. coli* green bar).

### Antifungal activity of treated samples

**Figure 12:**
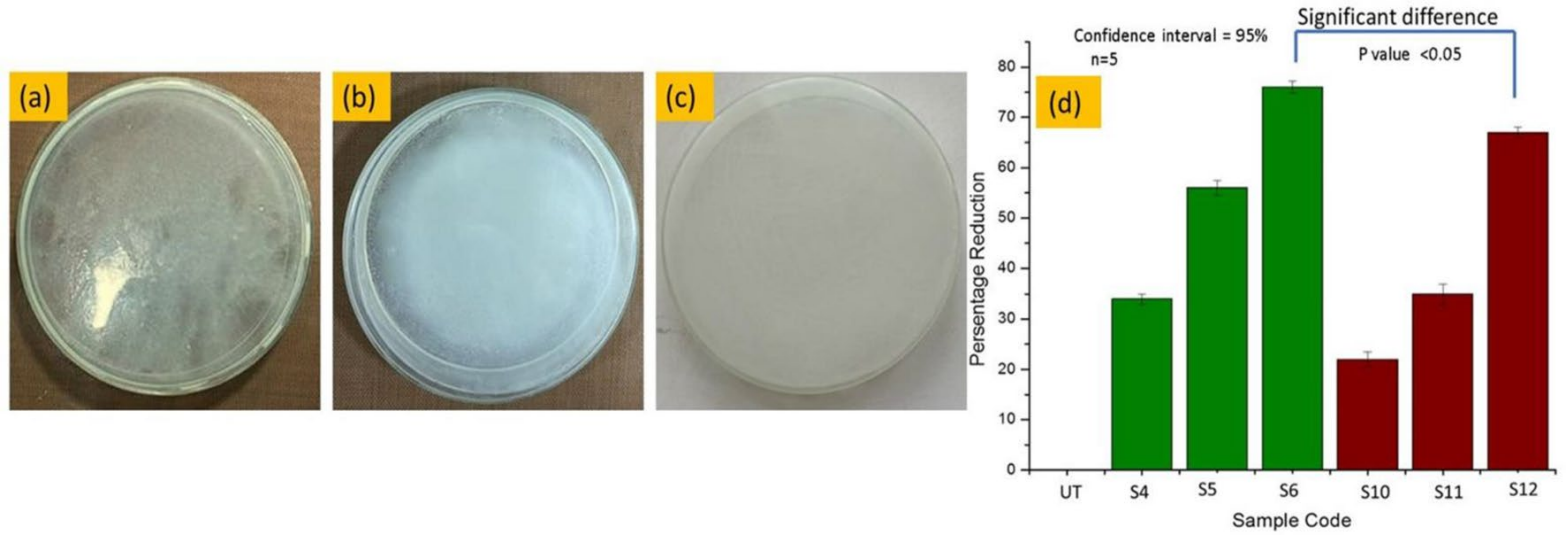
Images shows fungus growth against (**a**) silver particles coated samples, (**b**) against copper particles coated samples, (**c**) raw cotton and (**d**) percentage reduction in fungal spore germination for each fabric specimen.

In order to assess the effectiveness of various fabric samples against the *A. niger* fungus, the AATCC-100 method was utilized in this study. Figure 12a–d showed the results related to fungus growth against each particle coated sample and percentage reduction in fungal spore germination for each fabric specimen. However, it was observed that fabrics with particle coatings were better in combating fungi when compared to untreated samples**.** Silver coated fabrics had the greatest inhibition of fungal growth among the particles-coated samples with antifungal effectiveness of approximately 77%. In fact, the present study showed better antipathogenic properties of silver particles overall. The statement can be further justified from a related study; where green synthesized silver particles showed almost the same reduction in percentage of fungus^36^. The group S6 contains the Ag-NPs coated fabric samples showing the reduction percentage of fungal activity values against *A. niger,* whereas group S12 contains the Cu-NPs coated fabric samples showing the reduction percentage of fungal activity values. While comparing the reduction percentage of fungal activity values against *A. niger* between S6 and S12. The tail of error bar of silver coated S6 sample group is not coinciding with the head of the error bar copper coated S9 sample. It means there is significant difference between two groups, which implies that silver and copper particles coated samples have reduction percentage of fungal activity values. The *P* value between these two groups was observed as 0.013 which is *P* < 0.05. Hence the *P* value was less than 0.05 which means there is significant difference between silver coated S6 sample group (percentage of fungal activity values against *A. niger*) and copper coated sample S12.

### Antiviral effectiveness

**Figure 13:**
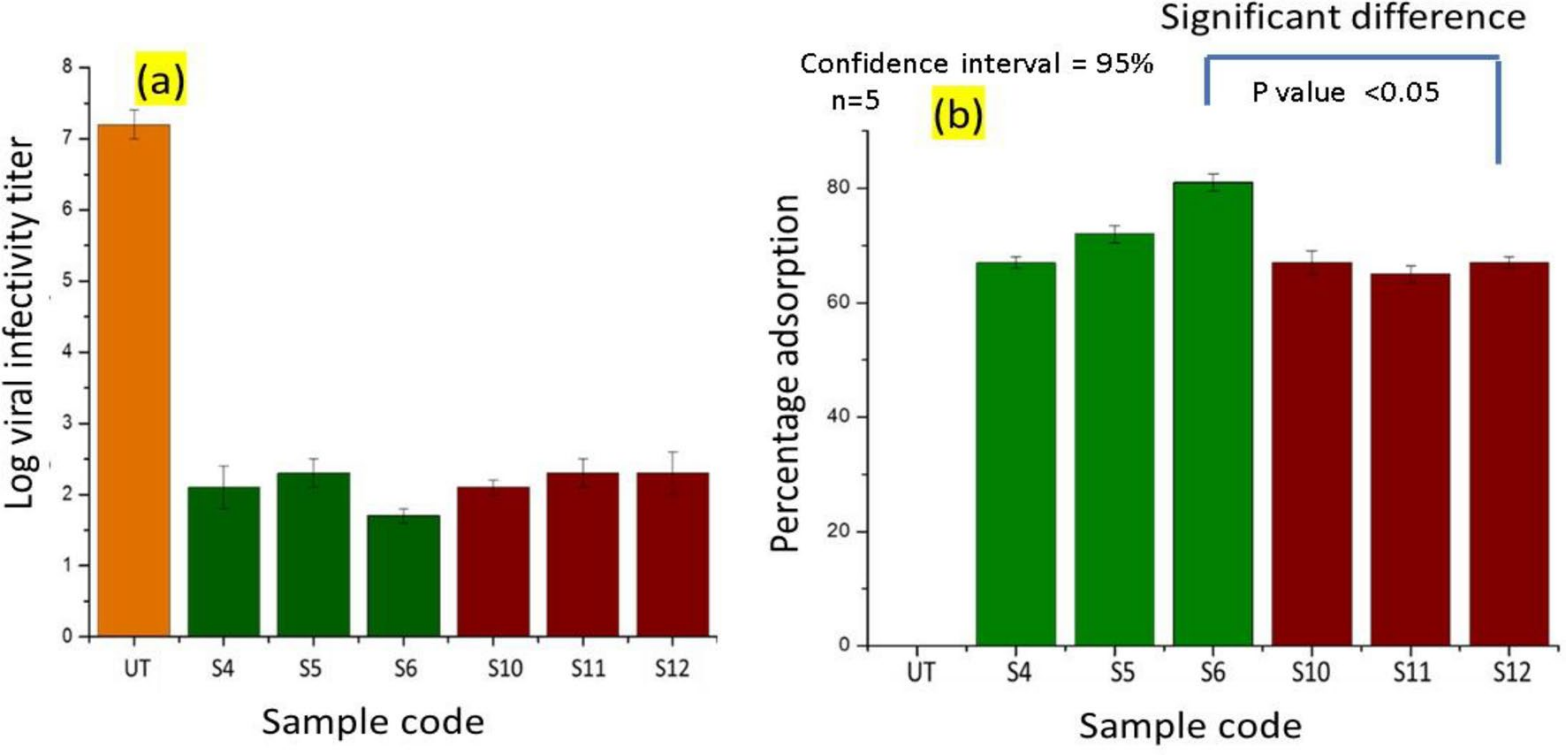
Reduction in viral infectivity titer (**a**) and percentage adsorption (**b**) calculated from viral infectivity at a contact time of 0 and 60 min.

The Behrens and Karber method was used to measure the antiviral effectiveness. Starting with initial viral titer of infectivity to determine the decrease in viral titer for coronavirus. The viral infectivity titer log is shown in Fig. 13 for both 0 h and 6 min. Overall, it showed that all treated samples (S4-S6 and S10-S12) with nanoparticles had sharply reduced viral infectious titer more than double as compared to untreated samples. However, there was no considerable difference of the titer amount in samples treated with either Ag-NPs or Cu-NPs. It indicates both silver and copper nanoparticles are almost equally effective in reducing viral infection in tested cell lines.

One possible mechanism for the suppression of viruses and the antiviral effects seen involves the interaction between particles and glycoproteins on the viral surface. In a recent research silver particle coated fabric were fabricated by photo deposition method. The couple effect of Ag^0^/Ag^+^ redox active agent exhibits 97% viral reduction specific to SARS-CoV-2^42^. The group S6 contains the Ag-NPs coated fabric samples showing the virus adsorption in percentage by nanoparticles, whereas group S12 contains the Cu-NPs coated fabric samples showing the virus adsorption in percentage by nanoparticles. While comparing the virus adsorption in percentage between S6 and S12. The tail of error bar of silver coated S6 sample group is not coinciding with the head of the error bar copper coated S9 sample. It means there is significant difference between two groups, which implies that silver and copper particles coated samples have significant difference in virus adsorption in percentage. The *P* value between these two groups was observed as 0.03 which is *P* < 0.05. Hence the *P* value was less than 0.05 which means there is significant difference between silver coated S6 sample group and copper coated sample S12.

## Conclusion

The current research employed sustainable, inexpensive and eco-friendly method to synthesized two different types of nanoparticles. In present study the phytochemical analysis of the peels of *Citrus sinensis* revealed the phenolic contents (rich in phenols and flavonoids), served as reducing and as a dispersing agent during the green synthesis of metal nanoparticles. The study was conducted to reveal the antagonistic (in vivo and in vitro) potential of synthesized nanoparticles against plant pathogenic bacteria (*Pectobacterium carotovorum*) and pathogens effective against humans (*E. coli*, *S. aureus*) was studied.

A total of 12 samples were prepared, (S1 to S6 with silver particles and S7 to S12 with copper particles) with different concentration. The prepared particles were coated on potato slices and cotton bandages. It was observed that the silver-coated samples S3 (1 g of particles on potato slices) and S6 (1 g of particles on fabric) had a higher ZOI than copper particles coated samples S9 (1 g on potato slices) and S12 (1 g of particles on fabric) samples. The results were also justified statistically, where the significant difference (as *P* < 0.05) between the two groups (of silver and copper coated potato samples) S3 and S9, and between the groups (of silver and copper coated fabric samples) S6 and S12 was found.

During the In-vivo analysis of particles over the potato slices. The bacterial strains of (*P. carotovorum*) showed almost zero infection against silver particles coated potato sample S3, while the potato slice coated with copper particles S9 showed very slight zone of infection, However, the clear and large zone of infection was seen on uncoated potato slice. Moreover, during in vitro analysis (antibacterial, antiviral, and antifungal) of prepared bandages the silver particles coated fabrics S6 with higher concentration (1 g) showed the 78% and 84% of antifungal and antiviral activity respectively. It means, the waste of peels contains quite effective bioactive agents that can be used against diverse types of pathogens. The surface morphology and existence of metals were analyzed by SEM, dynamic light scattering, EDS and XRD. The durability and retention of particles over the fabric surface was also analyzed by sever washing cycles. The developed fabrics can be effectively used to fabricate bioactive sportswear or active wears, bioactive compression garments as well as winter gloves, and compression bandages.

## Data Availability

All data generated or analysed during this study are included in this published article [and its supplementary information files].
