# Author Correction: Green synthesis of metal nanoparticles and study their anti-pathogenic properties against pathogens effect on plants and animals

**DOI:** 10.1038/s41598-024-63268-5

**Published:** 2024-05-29

**Authors:** Osama Usman, Mirza Muhammad Mohsin Baig, Mujtaba Ikram, Tehreem Iqbal, Saharin Islam, Wajid Syed, Mahmood Basil A. Al-Rawi, Misbah Naseem

**Affiliations:** 1 Department of Physics, University of Lahore, Lahore, Pakistan; 2 Department of Physics, University of Barcelona, Barcelona, Spain; 3 Institute of Chemical Engineering and Technology (ICET), University of Punjab, Lahore, Pakistan; 4 Department of Pharmaceutical Sciences, North South University, Dhaka, Bangladesh; 5 Department of Clinical Pharmacy, College of Pharmacy, King Saud University, 11451 Riyadh, Saudi Arabia; 6 Department of Optometry, College of Applied Medical Sciences, King Saud University, Riyadh, Saudi Arabia; 7 Department Chemical Engineering, Beijing Institute of Technology, Beijing, China

Correction to: *Scientific Reports* 10.1038/s41598-024-61920-8, published online 18 May 2024

The original version of this Article contained an error in the Funding section.

“The authors of this study extend their appreciation to the Research Supporting Project, King Saud University, Riyadh, Saudi Arabia, for supporting this study (RSP-2023/378) and for funding this work. Funded by Research Supporting Project, King Saud University, Riyadh Saudi Arabi with the following Grant Number RSP2024R378).”

now reads:

“The authors of this study extend their appreciation to the Research Supporting Project, King Saud University, Riyadh, Saudi Arabia, for supporting this study with the following Grant Number (RSP2024R378).”

The original Article has been corrected.

